# Alzheimer’s Disease Detection Using Combined EEG Source Connectivity and Microstate Features

**DOI:** 10.3390/brainsci16080856

**Published:** 2026-08-13

**Authors:** Lu Huang, Zheng Hu, Zhengnan Zhang, Yunyuan Gao

**Affiliations:** 1 HDU-ITMO Joint Institute, Hangzhou Dianzi University, Hangzhou 310005, China; 252320050@hdu.edu.cn (L.H.); 232320069@hdu.edu.cn (Z.Z.); 2 School of Automation, Hangzhou Dianzi University, Hangzhou 310005, China; 252060196@hdu.edu.cn

**Keywords:** Alzheimer’s disease (AD), EEG source localization (ESL), weighted phase lag index (wPLI), microstate

## Abstract

**Highlights:**

- A multi-domain feature fusion algorithm (SMMS) is proposed for EEG-based Alzheimer’s disease detection, combining source connectivity and microstate features.
- A unified microstate template is introduced to standardize analysis, enabling robust feature extraction and enhanced diagnostic performance under strict subject-independent validation.
- Alpha-band functional connectivity and microstate parameters are identified as exploratory candidate features for AD assessment.

**Abstract:**

**Background/Objectives:** Electroencephalography (EEG) connectivity and microstate analysis have shown great potential for Alzheimer’s disease (AD) diagnosis; however, their clinical application remains limited by the low spatial resolution of EEG and the lack of standardized microstate analysis. To address these challenges, this study proposes a multi-domain feature fusion framework, namely Source-localized Microstate and Multi-frequency Synchronization (SMMS), which integrates EEG source localization (ESL)-based weighted phase lag index (wPLI) functional connectivity with EEG microstate features. **Methods:** Specifically, ESL was employed to improve the spatial resolution of EEG signals for constructing functional connectivity matrices, while multi-frequency-band wPLI features were extracted to characterize functional synchronization among cortical regions. Meanwhile, EEG microstate features were utilized to capture the temporal dynamics of brain functional states. The proposed framework was evaluated on a public OpenNeuro dataset comprising 36 AD patients, 23 frontotemporal dementia (FTD) patients, and 29 healthy controls (HCs), as well as an additional clinical dataset collected from 48 AD patients at Sir Run Run Shaw Hospital, Hangzhou, China. **Results:** Experimental results showed that the proposed SMMS framework achieved high classification performance on both datasets. **Conclusions:** These findings demonstrate its effectiveness for EEG-based Alzheimer’s disease diagnosis.

## 1. Introduction

Alzheimer’s Disease (AD) is a neurodegenerative disease characterized by progressive cognitive decline [1]. The main clinical manifestations of AD include memory loss (especially short-term memory), impaired language function, reduced judgment and decision-making skills, and behavioral and emotional changes [2]. Patients usually present with mild cognitive impairment in the early stages of the disease, and as the disease progresses, progress to moderate and severe dementia, and eventually lose the ability to care for themselves [3]. AD not only seriously affects the quality of life of patients but also creates a serious economic and care burden on families and society. Although there is currently no cure, early diagnosis and targeted treatment are essential to delay the progression of Alzheimer’s and improve patients’ quality of life.

Existing diagnostic techniques mainly include magnetic resonance imaging (MRI) and positron emission tomography (PET) [4], but these techniques have certain limitations in terms of cost, accessibility and patient comfort, and their low temporal resolution makes it impossible to capture subtle dynamic changes [5]. With its millisecond temporal resolution, EEG can capture subtle changes in neural activity, which is crucial for the diagnosis of AD [6]. In addition, the non-invasive characteristics of EEG ensure the safety of patients [7], and compared with other imaging techniques, EEG is less expensive, portable, and widely applicable [8]. From these points, EEG offers a high-potential avenue for AD research and clinical diagnosis. There are many methods for EEG-based AD research, among which microstate and functional connectivity matrix are important directions in current research [9,10].

EEG microstate is a method of analyzing EEG signals that segment brain electrical activity into short-lived stable patterns, usually lasting tens to hundreds of milliseconds [11]. Each state is temporally stable, while spatially exhibiting a specific potential distribution that may correspond to a specific functional brain activity [12]. Large-scale EEG microstate data analysis also enables the discovery of specific patterns that can be used as biomarkers for AD for early screening and preventive diagnosis [13]. By analyzing the microstate features in the EEG, abnormal brain activity in AD patients can be found. Yan et al. conducted an in-depth analysis of microstates by collecting resting-state EEG data from both groups [14]. The findings revealed that, compared to the healthy controls (HC), AD patients exhibited significantly longer durations in microstates C and D, while the occurrence of microstate B was reduced. Li et al. proposed a novel method for constructing brain networks based on microstate sequences [15]. The findings indicated that the parameters for microstate class B were higher in the mild cognitive impairment (MCI) group compared to the HC group. Additionally, the stability ofbrain connectivity across most channels in both the MCI and AD groups was observed to be lower than that of the HC group.

In addition to the characteristics of multi-channel EEG signals, the analysis of connectivity between channels is also crucial for understanding the differences in brain function among different groups. EEG connectivity analysis has an important application in the research and diagnosis of AD [16]. It can help to detect the abnormalities of brain networks in AD patients, such as lower functional connectivity on certain frequency bands, which can be used as potential biomarkers for early diagnosis and risk assessment [17]. Si et al.’s study reported an 81.1% diagnostic accuracy for frontotemporal dementia (FTD) and AD by constructing a frequency-based multi-layer resting-state functional connectivity matrix [18]. Sebastian et al.’s study explores changes in functional connectivity during sleep in patients with AD and its relationship with network hyperexcitability and cognitive function [19]. The results showed that AD participants exhibit increased gamma connectivity during Stage 2 sleep (N2), which is associated with longitudinal cognitive decline. Although EEG microstate and functional networks have shown great potential in AD research, there are still some challenges in the current research. Firstly, the complex signal processing and analyzing EEG microstates have not been fully standardized, leading to differences and lack of consistency in the results between different studies [20]. In addition, the spatial resolution of traditional EEG is limited, which makes it difficult to accurately locate and distinguish the activity of different brain regions, thus affecting the establishment and analysis of brain functional networks [21]. Both deficiencies can affect the accuracy in AD diagnosis. Ge et al. proposed an EEG-based framework for predicting the conversion from amnestic MCI to AD using longitudinal EEG recordings [22]. Yuan et al. summarized quantitative EEG biomarkers, including spectral features, functional connectivity, and microstates, and emphasized their diagnostic potential for AD and MCI [23]. Chetty et al. further demonstrated that EEG biomarkers in prodromal AD exhibit significant abnormalities in synchronization and functional connectivity [24]. In addition, Jiao et al. employed EEG biomarkers combined with machine learning algorithms for HC/MCI/AD classification and achieved promising diagnostic performance in large-scale cohorts [25]. To address the aforementioned issues, a multi-domain feature fusion algorithm is proposed, integrating the multi-frequency band brain functional matrix following EEG source localization (ESL) with EEG microstates (SMMS), aiming for the efficient diagnosis of AD patients. Specifically, ESL was used to improve the spatial resolution of EEG signals, and multi-frequency weighted phase-lag index (wPLI) matrices were extracted to enhance the analysis ability of brain functional networks. Additionally, a unified microstate template was introduced to address the lack of standardization in microstate analysis. Table 1 compares representative studies on EEG-based Alzheimer’s disease diagnosis.

## 2. Materials and Methods

**Figure 1:**
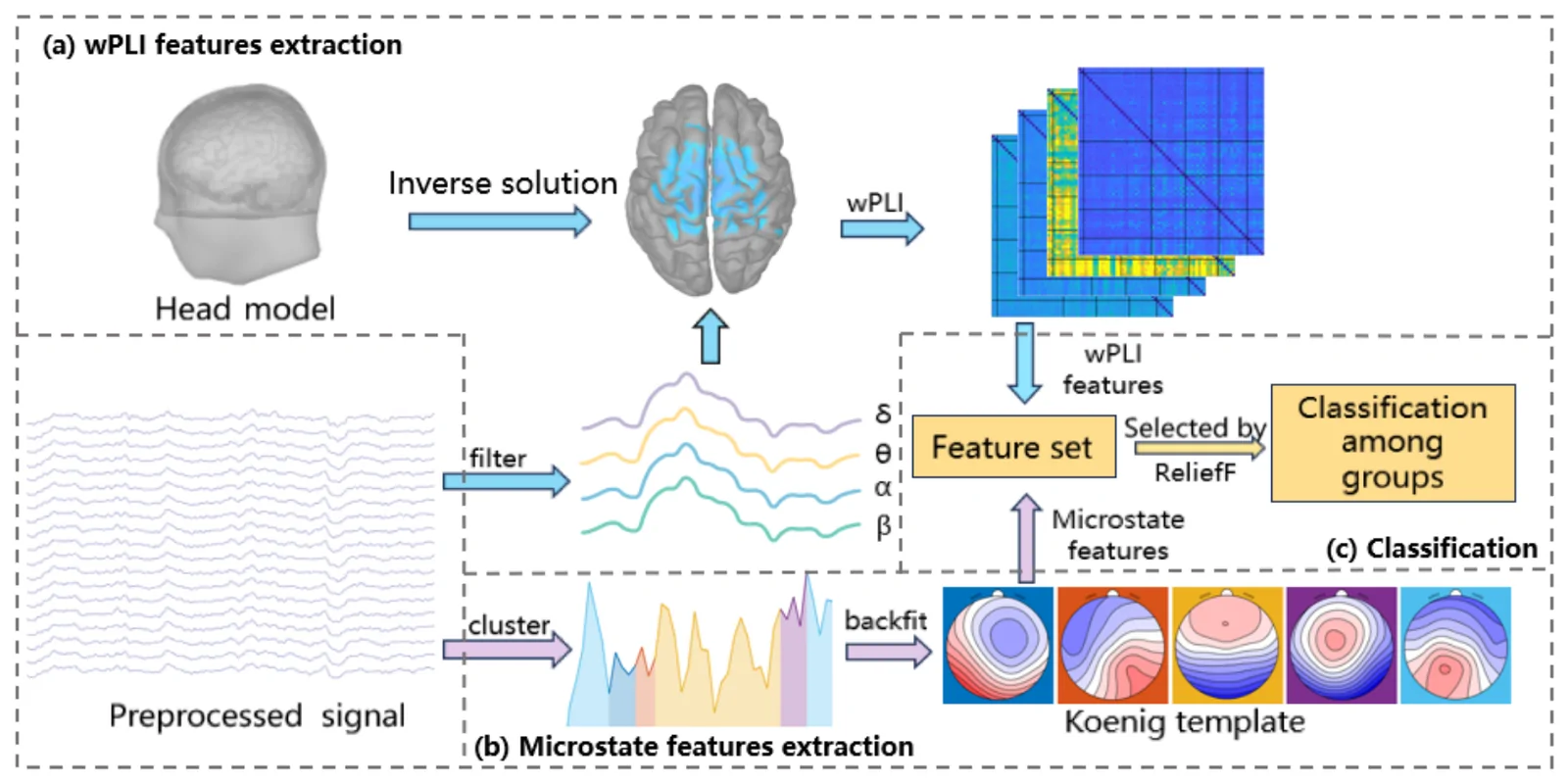
Algorithmic framework of SMMS. (**a**) wPLI features extraction: Separate the signal into distinct frequency bands, with sLORETA used for ESL, and extract average intensity from the wPLI matrix. (**b**) Microstate features extraction: The EEG’s global field power was clustered into five microstates using k-means, followed by the extraction of features. (**c**) Classification: Features were fused, selected with ReliefF, and classified using k-NN.

In this section, two datasets are presented: one is a publicly available dataset from OpenNeuro, and the other one is a self-collected dataset in collaboration with Sir Run Run Shaw Hospital, Hangzhou, China. The flowchart of the whole SMMS framework is shown in Figure 1. Initially, EEG signals were preprocessed with filtering, artifact correction, and segmentation, resulting in specific segment counts for different groups across two datasets with consistent electrodes. Then signals underwent ESL to enhance spatial resolution [26], and the multi-frequency wPLI matrices were extracted to achieve more precise brain activity analysis and efficient combination of spatial and frequency domains [27]. Simultaneously, the preprocessed EEG signals underwent clustering to identify microstates and temporal features were derived from them. To address the lack of standardized protocols, a unified microstate template is employed to enable standardized comparisons. Finally, features selected by ReliefF were used to classify different groups.

### 2.1. Datasets

#### 2.1.1. Dataset I

This dataset derives from an open dataset, which includes resting-state data from 88 subjects, comprising 36 AD patients, 23 FTD patients, and 29 HC [28]. All subjects were assessed for cognitive and neuropsychological status using the Mini-Mental State Examination (MMSE) [29]. The total score on the MMSE ranges from 0 to 30, with higher scores indicating better cognitive function and lower scores indicating more pronounced cognitive decline. The average MMSE score for the AD group was 17.75 ± 4.5, and for the FTD group was 22.17 ± 8.22, while the average MMSE score for the healthy control group remained at 30. The EEG recording included signals from 19 scalp electrodes (Fp1, Fp2, F7, F3, Fz, F4, F8, T3, C3, Cz, C4, T4, T5, P3, Pz, P4, T6, O1, and O2) and 2 reference electrodes (A1, A2), arranged according to the 10–20 international system. Each recording was performed according to clinical protocols. Before each recording, the impedance of each electrode was kept below 5 kΩ. The sampling rate was set to 500 Hz, and the subject was required to sit in a chair with their eyes closed. The received recordings contained specific amplifier parameters: sensitivity was set to 10 uV/mm, time constant was 0.3 s, and the high-pass filter was set to 70 Hz. The average recording time for the AD group was 13.5 min (range [5.1, 21.3] min), for the FTD group it was 12 min (range [7.9, 16.9] min), and for the HC group it was 13.8 min (range [12.5, 16.5] min). In total, 485.5 min of recordings were obtained for the AD group, 276.5 min for the FTD group, and 402 min for the HC group.

#### 2.1.2. Dataset II

The dataset was collected from June 2022 to September 2022 at the Department of Mental Health, Sir Run Run Shaw Hospital, Hangzhou, China. Patients were resource participants and signed informed consent at the time of enrollment. The study was approved by the Hospital Ethics Committee (Approval No.: 20200818-8). All data processing was performed in compliance with international and local laws and regulations on data protection. A total of 48 patients with a definite diagnosis of AD were included in this dataset. The average MMSE score was 18.83 ± 5.2. The EEG recording included signals from 64 scalp electrodes arranged according to the 10-10 international system. Each recording was performed with the same clinical protocol as Dataset I. The average recording time was 6.9 min (range [4.8, 11.6] min). In total, 332.6 min of recordings were obtained for the Dataset II AD group. Table 2 shows the demographic characteristics of the two datasets.

### 2.2. EEG Preprocessing

The resting-state EEG signals were preprocessed as follows. Firstly, a Butterworth 1–30 Hz bandpass filter was applied, and the signals were re-referenced to A1–A2. Then, the Artifact Subspace Reconstruction (ASR) method was employed to correct EEG artifacts. In ASR, the burst correction threshold was set to 17 times the calibration standard deviation, which is a relatively conservative threshold commonly used to suppress high-amplitude transient artifacts while avoiding excessive removal of valid EEG activity in clinical recordings [30]. Abnormal data segments exceeding this threshold within a maximum acceptable 0.5 s window were identified and corrected. Subsequently, Independent Component Analysis (ICA) was performed to remove physiological artifacts. Specifically, ICLabel was used to automatically identify eye movement and muscle-related artifact components, followed by manual inspection to ensure artifact removal accuracy [31]. Finally, the data were segmented into 30s epochs.

After preprocessing, in the Dataset I, the number of segments was 469 for the AD group, 264 for the FTD group and 388 for the HC group. In the Dataset II, the number of segments was 324 for the AD group. Meanwhile, in order to maintain the consistency of specifications, only the same 19 electrodes as in Dataset I were retained.

### 2.3. EEG Source Localization (ESL)

EEG source localization (ESL) is a neuroimaging technique that estimates the location, orientation, and intensity of cortical source signals from EEG signals [32]. This process involves solving both forward and inverse problems.

For the forward problem [33], the cortical source signal must traverse the skull and scalp to be captured by EEG electrodes, necessitating a head conduction model. In this study, the Colin27 head template was used as the head model for all subjects [34]. A three-layer model comprising the cortex, skull, and scalp was constructed using the boundary element method (BEM) [35], and the lead-field matrix L was calculated using OpenMEEG v2.4.2 software [36].

For the inverse problem [37], which involves calculating cortical source signals from scalp EEG signals, the large number of source signals compared to the number of electrodes results in an ill-posed problem. In this paper, sLORETA was used to solve the inverse problem.

In the sLORETA method [38], the first step is to establish the following relationship between the lead-field matrix L and the electrode measurement data V:

(1) V=LJ+n

where J represents the source current density, and n is noise. Based on this model, sLORETA solves for the current density by minimizing the error function:

(2) $$\hat{J}=\arg\min_{J}\left\{{∥V-\mathrm{LJ}∥}^{2}+\lambda {∥J∥}^{2}\right\}$$

where λ is a regularization parameter that controls the stability and accuracy of the solution. Subsequently, sLORETA applies standardization to the estimated current density:

(3) $${\hat{J}}^{\prime }=\frac{\hat{J}}{\sigma (J)}$$

The specific standardization expression is

(4) $${\hat{J}}_{\mathrm{sLORETA}}={\left(L^{T}L+\lambda I\right)}^{-1}L^{T}V/\sqrt{\mathrm{diag}\left({\left(L^{T}L+\lambda I\right)}^{-1}\right)}$$

In this study, the Shape Variability Registration (SVreg) method was used to segment the brain structures [39], resulting in 66 regions of interest (ROIs) [40].

### 2.4. Weighted Phase Lag Index (wPLI)

wPLI is a metric used to analyze phase synchronization between brain regions using EEG or magnetoencephalography (MEG) data [41]. Compared to the traditional Phase Lag Index (PLI) [42]. wPLI reduces the impact of spurious phase synchronization signals through weighted processing, thus providing a more accurate reflection of actual functional connectivity between brain regions. The calculation steps for wPLI include signal preprocessing, calculating the phase difference, normalizing the phase difference weights, and calculating the overall wPLI value. The core formula is given by

(5) wPLI=|〈Im(Cij(t))〉|〈|Im(Cij(t))|〉

where Im(Cij(t))=Im(Sij(t))|Sij(t)| represents the normalized imaginary part of the cross-spectrum between signals *i* and *j* at time *t*, with $S_{ij}\left(t\right)$ being the time-averaged cross-spectral density of signals $x_{i}\left(t\right)$ and $x_{j}\left(t\right)$. The notation 〈·〉 denotes averaging over time. The wPLI value ranges between 0 and 1, where 0 indicates no phase synchronization and 1 indicates complete synchronization between the signals.

In this study, EEG signals after ESL were divided into delta (1–4 Hz), theta (4–8 Hz), alpha (8–13 Hz), and beta (13–30 Hz). The wPLI matrix of 66 × 66 size was then extracted for each rhythmic wave, where each element represented the strength of functional connectivity between the two ROIs. Since the wPLI matrix is symmetric, i.e., wPLI[i][j] = wPLI[j][i], the upper and lower triangular parts contain redundant information. Therefore, only the upper triangular elements were retained for subsequent feature extraction and submatrix averaging. In order to analyze the brain network more clearly, the matrix was summarized into 6 × 6 submatrices according to the predefined brain partitioning scheme, which included insula with 2 ROIs, frontal with 24 ROIs, parietal with 12 ROIs, temporal with 12 ROIs, occipital with 10 ROIs, and limbic with 6 ROIs.

### 2.5. Microstate

EEG microstate refers to a transient, stable pattern of electrical activity observed in electroencephalogram (EEG) signals, which helps to reveal the evolution of the functional state of the brain by using temporal domain information from the dynamic perspective of brain electrical activity.

In this study, for each group and subject, the global field power (GFP) of all electrodes was extracted [43], and only the topography at the instantaneous value of GFP was selected for clustering to ensure a high signal-to-noise ratio.

The modified k-means algorithm is used as the clustering algorithm [44]. Since the results of the k-means algorithm depend on randomly chosen starting conditions, it may lead to each clustering that does not always produce a globally optimal solution. Therefore, the range of k was set to 2 to 8, while 20 random restarts were set to improve reliability.

**Figure 2:**
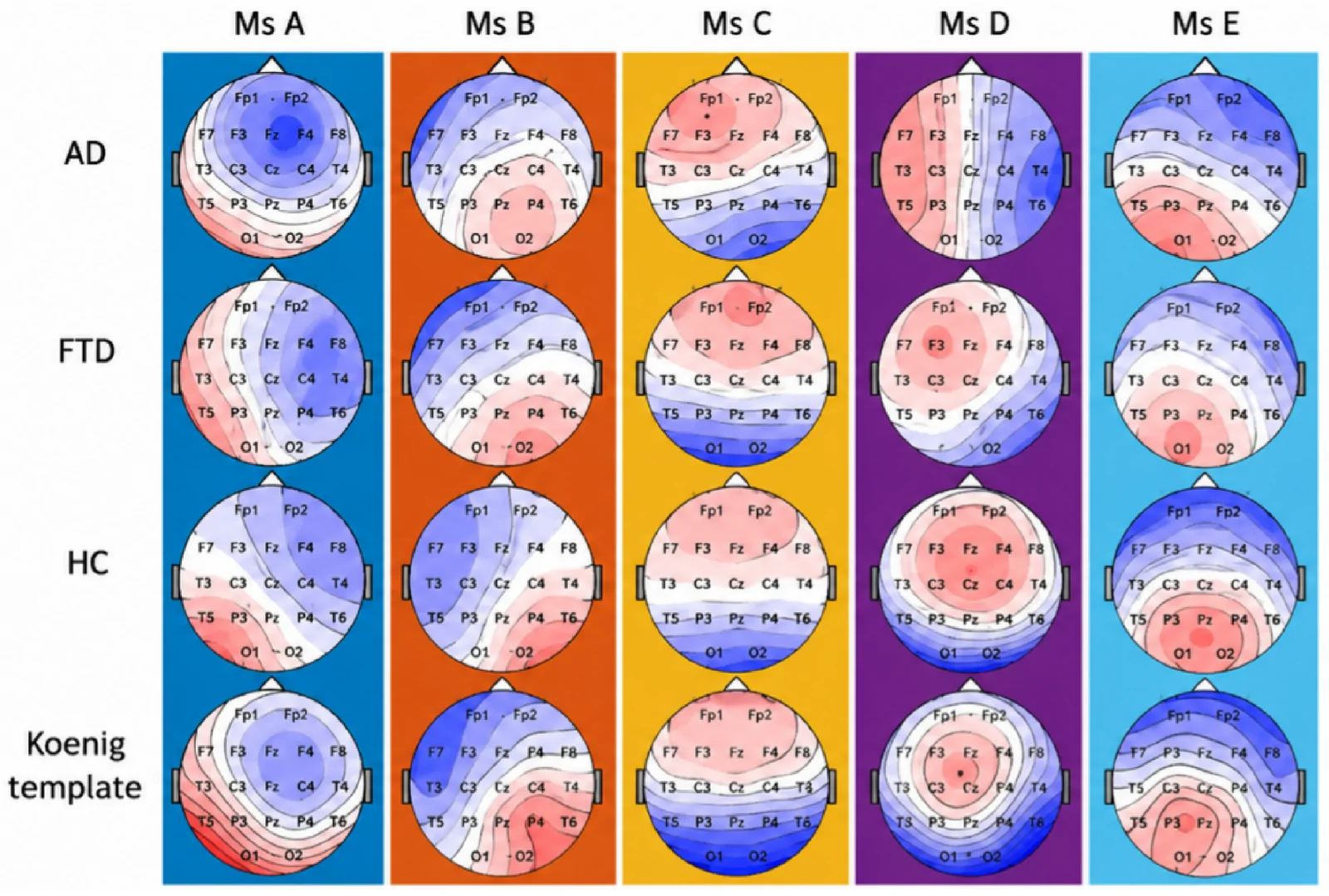
Average EEG microstate templates of the AD, FTD, and HC groups together with the Koenig template. Red and blue colors represent positive and negative scalp potentials, respectively.

Since there is now no clear regulation on the number of categories for microstates, the number of microstate classes for each group was fixed at 5 [20]. The average microstate templates of each group were calculated separately, and it was found that the average maps among groups were very similar except for the fourth microstate of AD group. It has been proven that if the topographic features of the average template map are sufficiently similar to those of the published template and have high spatial correlation, they can be considered to be functionally identified as similar [13]. At the same time, for the sake of conservative statistical analysis, the feature extraction of microstates should always be based on a single common average template [45]. Figure 2 shows the average microstate templates of the AD group, FTD group, HC group and the template of Koenig.

For each reverse-matched microstate record, four features were extracted, including duration, coverage, occurrence and state transition probability matrix. Duration rep-resents the average length of time a microstate lasted. Coverage represents the proportion of the total record covered by each microstate. Occurrence represents the number of times that microstate occurred per second. The transition probability matrix represents the probability of transition from one microstate to another.

### 2.6. Feature Selection

For each subject, the wPLI submatrices covering 6 brain regions across four frequency bands were utilized. The average value of each submatrix was extracted as a feature. In particular, submatrices containing diagonal elements were stripped of the diagonal elements before being averaged.

Given the symmetrical property of wPLI, that is, for any *i* and *j*, wPLI[i][j]=wPLI[j][i], the size of the wPLI feature part is $\left(\frac{6\times 6-6}{2}+6\right)\times 4=84$.

On the other hand, each of the 5 microstates has its own coverage, duration, and occurrence. Moreover, the state transition matrix assumes no transition within the same state. Thus, the microstate part contains 5×3+5×5−5=35 features. Therefore, each subject has a total of 84+35=119 features.

In this paper, feature selection via ReliefF and feature normalization (Z-score) were strictly performed within each training fold during the subject-level cross-validation process. Feature ranks were determined solely based on the training subjects in each fold [46]. The ReliefF algorithm starts by initializing a weight vector for each feature, usually with all weights set to zero. It then randomly selects an instance from the training set and finds the *k* nearest neighbors from the same class (nearest hits) and the *k* nearest neighbors from each of the different classes (nearest misses). For each feature, the weight is updated based on how well it distinguishes between the instance and its neighbors. Specifically, the weight is increased if the feature value differs more between the instance and misses than between the instance and hits, and decreased otherwise. This process is repeated for a predefined number of iterations or until convergence. The resulting weights represent the importance of each feature for distinguishing between classes. Herein, the number of *k* nearest neighbors was 10. The detailed composition of extracted features before and after feature selection is summarized in Table 3.

### 2.7. Classification

To evaluate the effectiveness of the proposed SMMS framework, several machine learning classifiers were employed, including K-Nearest Neighbors (KNN), Support Vector Machine (SVM), Decision Tree (DT), Linear Discriminant Analysis (LDA), and Naive Bayes (NB).

For KNN, the number of nearest neighbors was set to 10. For SVM, a Gaussian kernel function was used. For DT, the classifier was implemented using the default tree model in MATLAB R2024a. LDA and NB were used as additional baseline classifiers with default MATLAB implementations.

All extracted features were normalized before classification. Classification performance was first evaluated using 10-fold cross-validation. To further evaluate model generalization, an additional subject-independent validation experiment was conducted by ensuring that EEG recordings from the same subject were not simultaneously included in the training and testing sets. Accuracy, precision, recall, and F1-score were selected as the evaluation metrics and calculated as follows:

(6) $$\mathrm{Accuracy}=\frac{\mathrm{TP}+\mathrm{TN}}{\mathrm{TP}+\mathrm{TN}+\mathrm{FP}+\mathrm{FN}}$$

(7) $$\mathrm{Precision}=\frac{TP}{TP+FP}$$

(8) $$\mathrm{Recall}=\frac{TP}{TP+FN}$$

(9) $$F1\text{-}\mathrm{score}=2\times \frac{\mathrm{Precision}\times \mathrm{Recall}}{\mathrm{Precision}+\mathrm{Recall}}$$

where TP, TN, FP, and FN denote the numbers of true positives, true negatives, false positives, and false negatives, respectively. For each binary classification task (AD/FTD, AD/HC, and FTD/HC), the disease group listed first was defined as the positive class and the other group as the negative class. For the three-class classification task (AD/FTD/HC), the evaluation metrics were calculated using a one-versus-rest strategy for each class, and the reported precision, recall, and F1-score were obtained by macro-averaging across the three classes.

## 3. Results

### 3.1. Performance of SMMS in Dataset I

The classification performance of five machine learning algorithms, including KNN, Support Vector Machine (SVM), Naive Bayes, Linear Discriminant Analysis (LDA), and Decision Tree (DT), evaluated on four classification problems: AD vs. FTD, AD vs. HC, FTD vs. HC and AD vs. FTD vs. HC. To achieve a good balance between accuracy and computational resources, the optimal 100 features were selected by the ReliefF algorithm to complete the classification. Multiple evaluation metrics including accuracy, precision, recall, F1 score were calculated to evaluate these machine learning algorithms.

In Table 4, all classification results in Dataset I with SMMS using 10-fold cross-validation were given. Among all the models, the KNN algorithm performed exceptionally well in classification tasks involving AD/FTD, FTD/HC, and AD/FTD/HC, with accuracy, precision, recall, and F1-Score all close to or exceeding 0.97. This indicated that SMMS with KNN had a very high classification capability in handling these specific tasks, effectively distinguishing between different neurodegenerative disease groups and healthy populations. In comparison, other models like SVM also performed well in certain tasks but overall were slightly inferior to KNN. LDA, DT, and Naive Bayes showed relatively poorer performance, especially in multi-classification tasks, possibly due to their limitations in handling complex data structures.

### 3.2. Performance of SMMS in Dataset II

In order to evaluate the preliminary feasibility of cross-dataset feature portability in SMMS, the AD group data collected from Sir Run Run Shaw Hospital were analyzed. Since the relevant data of healthy subjects were not included in this dataset, the classification experiments were conducted between the healthy subjects in the Dataset I and the AD group in this dataset. Similarly, the ReliefF algorithm was used to select the optimal 100 features for classification. The results are shown in Table 4, and similarly, KNN obtains the optimal result with classification accuracy of 0.978, precision of 0.980, recall rate of 0.968, and F1 score of 0.974.

### 3.3. Ablation Experiments and Comparisons

In order to evaluate the contribution of each component in the proposed method, a series of ablation experiments were designed, which mainly include the wpli submatrix features, microstate features, and the influence of ESL on the wPLI feature results. In addition, the results were compared with four existing methods. The classification results and comparisons with the other four methods in DS1 are shown in Table 5.

- wPLI: wPLI matrix features without ESL, the number of features is $\frac{19\times 19-19}{2}\times 4=648$.
- S-wPLI: The 84 features of the wPLI submatrix part.
- MS: The 35 features of the microstate contain duration, coverage, occurrence, and state transition matrix.
- Fre-Networks [18]: The model constructs a frequency-based multi-layer resting state EEG network and extracts representative network features to achieve classification.
- MTRRP [15]: The model achieves classification by analyzing the pattern of recurrence rate changes with recurrence threshold in EEG and combining recurrence rate gradient and recurrence Hurst index.
- SVD [47]: The model uses sliding window and extracts singular value decomposition entropy (SVD) as feature, and uses KNN to realize classification.
- Vit-CNN [48]: The model achieved classification by fusing the dual-branch network structure of Convolutional Neural Network (CNN) and Visual Transformers (ViT).

As can be seen from Table 5, the results of S-wPLI are higher than those of wPLI in the four classification experiments, which proved that the addition of the ESL improved the spatial resolution of the signal, and could more accurately reflect the connectivity differences of different people’s brain activities.

In addition, it was found that when only microstate features or wpli features were used, the accuracy was only 85.9% and 83.7% in the three-classification experiments of AD, FTD and HC, respectively. However, when these two features were combined, the accuracy can reach 97.3% in the three-classification experiment. It has been demonstrated that microstate features, which reflect temporal or dynamic characteristics, and the wPLI matrix, which captures phase synchronization features, each provide unique insights into brain activity. By effectively integrating these different types of information, efficient discrimination among different populations can be achieved.

Moreover, the proposed method was compared with the latest four machine learning and deep learning methods. In the four classification experiments, SMMS achieved the best classification results.

## 4. Discussion

### 4.1. EEG Source Signal wPLI

**Figure 3:**
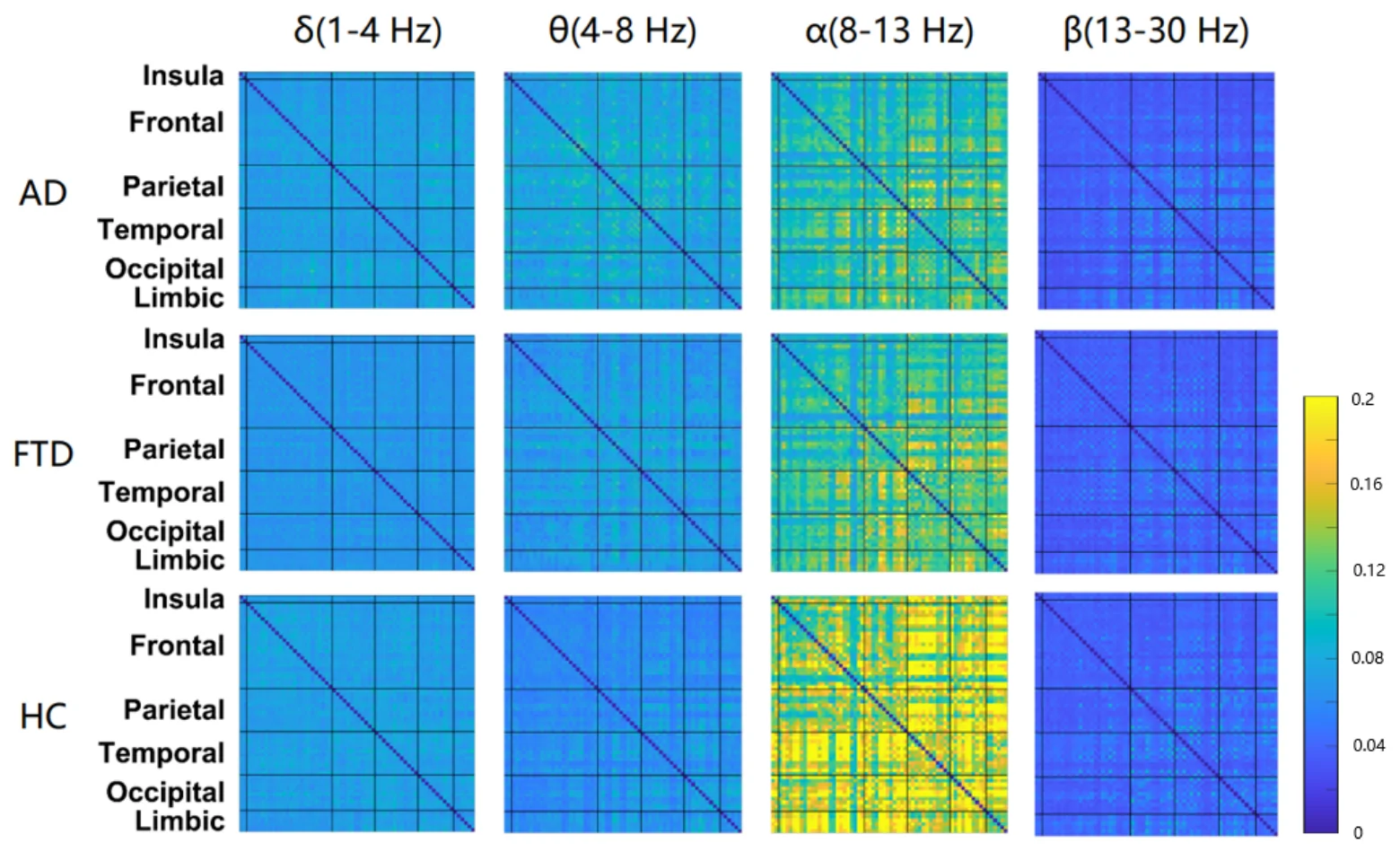
Averaged wPLI connectivity matrices across different frequency bands. Warmer colors indicate stronger functional connectivity, while cooler colors indicate weaker connectivity. The blue lines mark the boundary of each brain region, and the color bar represents the wPLI connectivity strength.

Figure 3 shows the wPLI matrix for each frequency band after ESL for AD, FTD, and HC groups in Dataset I. In general, the wPLI values of AD and FTD groups were very similar. The wPLI values of the three groups showed a similar distribution in different frequency bands, with the maximum value in alpha band (8–13 Hz) and the minimum value in beta band (13–30 Hz).

In addition, in the theta (4–8 Hz) region, the AD group had the largest wPLI value, followed by the FTD group and finally the HC group. While, in the beta band (13–30 Hz), the wPLI values of the AD group and FTD groups were significantly lower than those of the HC group. A large number of existing studies have proved that AD and FTD patients tend to have increased activity in theta and decreased activity in alpha. In this study, similar results were obtained in the wPLI matrix of the source signal after ESL.

Increased theta wave usually indicates that the brain is less efficient in processing information in resting state or active cognitive tasks. Moreover, theta wave is closely related to the temporal lobe mediation activity of the brain, and its increase may indicate the impairment of functions related to short-term memory and working memory [49]. Whereas alpha waves are usually associated with relaxation and resting states with eyes closed. Its reduction may reflect that AD patients are not sufficiently relaxed in the resting state. In addition, alpha wave attenuation is often associated with decreased information processing capacity of the brain at rest, which may affect an individual’s ability to perform complex cognitive tasks [50].

### 4.2. Microstate Parameters

The parameters of the microstate were compared between groups in Dataset I and are represented in Table 6 shows the specific parameters for five microstates in different groups. In addition, one-way ANOVA was used to analyze the parameters of each microstate across groups. If the *p*-value between groups is less than 0.0001, the parameter difference is considered to be significant. For duration, differences are observed with AD/FTD in state A, AD/FTD, AD/HC and FTD/HC in state B, AD/HC and FTD/HC in state C, and AD/FTD and FTD/HC in state E. In state B, the AD group had the longest duration, followed by FTD. In state C, the duration of HC group was significantly longer than that of the other two groups. However, in state E, the AD group had a significantly longer duration than the remaining two groups.

The differences are evident for coverage, with AD/HC and FTD/HC in state B, AD/HC and FTD/HC in state C, and AD/FTD and FTD/HC in state D. In state B, the coverage of HC group was significantly lower than that of the other two groups. However, in state C, the coverage of the HC group was significantly higher than that of the remaining two groups.

**Figure 4:**
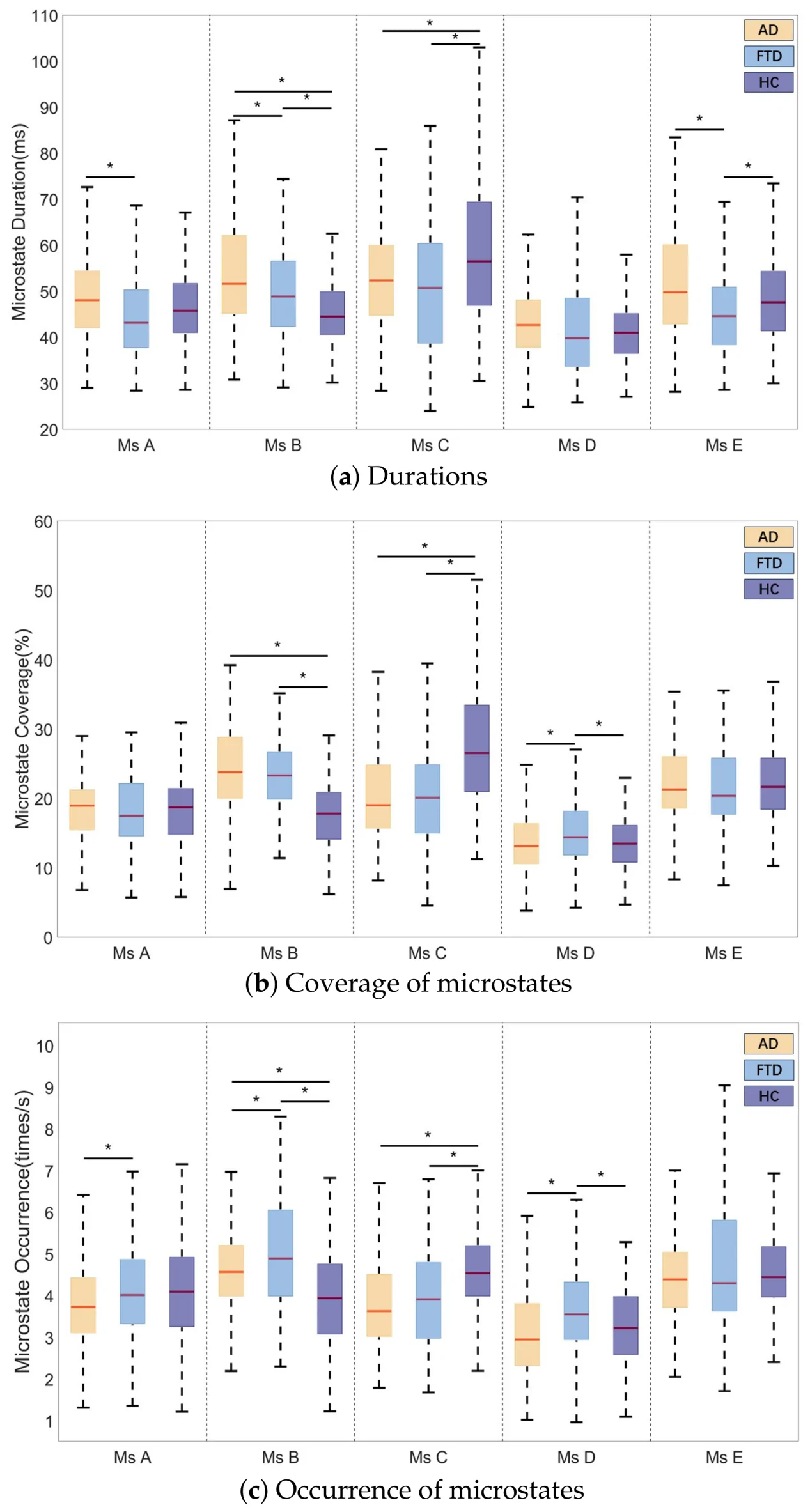
Microstate parameter box diagrams for AD, FTD, and HC groups: (**a**) duration of microstates, (**b**) coverage of microstates, and (**c**) occurrence of microstates. Group color designations are yellow for AD, blue for FTD, and purple for HC. Boxplots represent the median (red center line), the 25th and 75th percentiles (box bounds), and the 1.5 × IQR range (whiskers), with outliers displayed individually as separate points (^∗^ *p* <0.0001).

The differences are evident for occurrence, with AD/FTD observed in state A, AD/FTD, AD/HC and FTD/HC in state B, AD/HC and FTD/HC in state C, and AD/FTD and FTD/HC in state D. In state B, the occurrence of FTD was the highest followed by AD group, and in state C, the occurrence of HC group was significantly higher than that of the other two groups, while in state D, the occurrence of FTD was significantly higher than that of the other two groups. The distributions of duration, coverage, and occurrence across groups for each microstate are illustrated in Figure 4.

### 4.3. Microstate Transition Probability

In addition to the three parameters of the microstate including duration, coverage, and occurrence, transition probabilities between microstates were also extracted.

**Figure 5:**
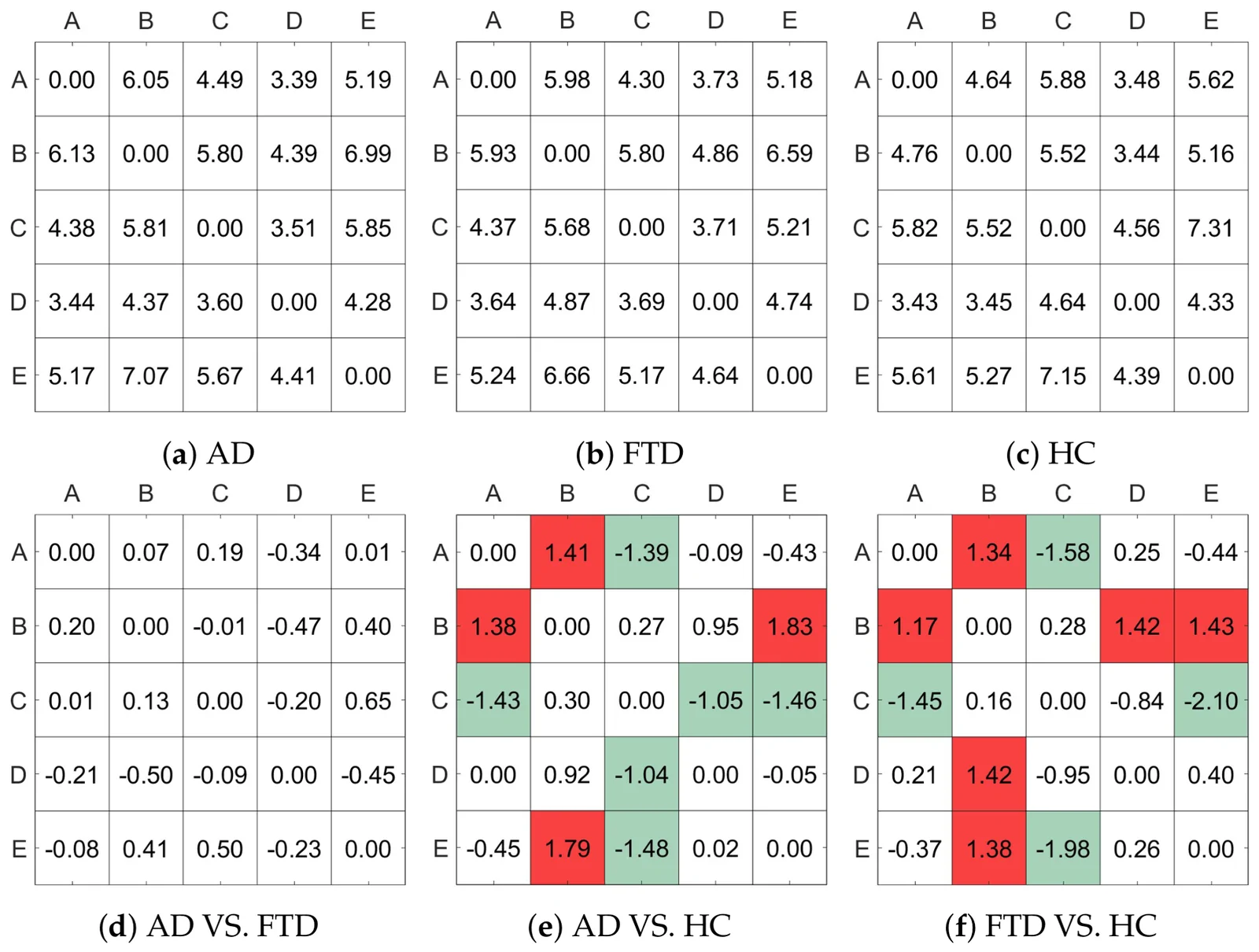
State transition matrices for the diagnostic groups: (**a**–**c**) represent state transition probability matrices for the AD, FTD, and HC groups, respectively. Panels (**d**–**f**) depict pairwise group differences (the former minus the latter). For descriptive visualization rather than inferential testing, cells with numerical differences greater than +1.0% or lower than −1.0% are highlighted in red and green, respectively.

Figure 5a–c represent the probabilities of transitions between states in the AD group, the FTD group, and the HC group, respectively. Figure 5d–f show the difference in transition probabilities between the different groups separately. The parts where the difference in transition probabilities is greater than plus or minus one are tabulated in red and green, respectively. The results show that the transition probability matrices of AD and FTD groups were very similar. However, there were significant differences between HC and these two groups. As a descriptive observation, cells with transition probability differences exceeding ±1.0% were highlighted (red for higher and green for lower transitions). Specifically, relative to the HC group, both AD and FTD groups displayed numerically lower mean transition probabilities between states A → C, C → D, and C → E, alongside higher mean transition probabilities between states A → B, B → D, and B → E. Note that these observed variations are presented descriptively to illustrate numerical trends, without formal statistical hypothesis testing.

### 4.4. Correlational Analysis Between Microstate Parameters and wPLI

**Figure 6:**
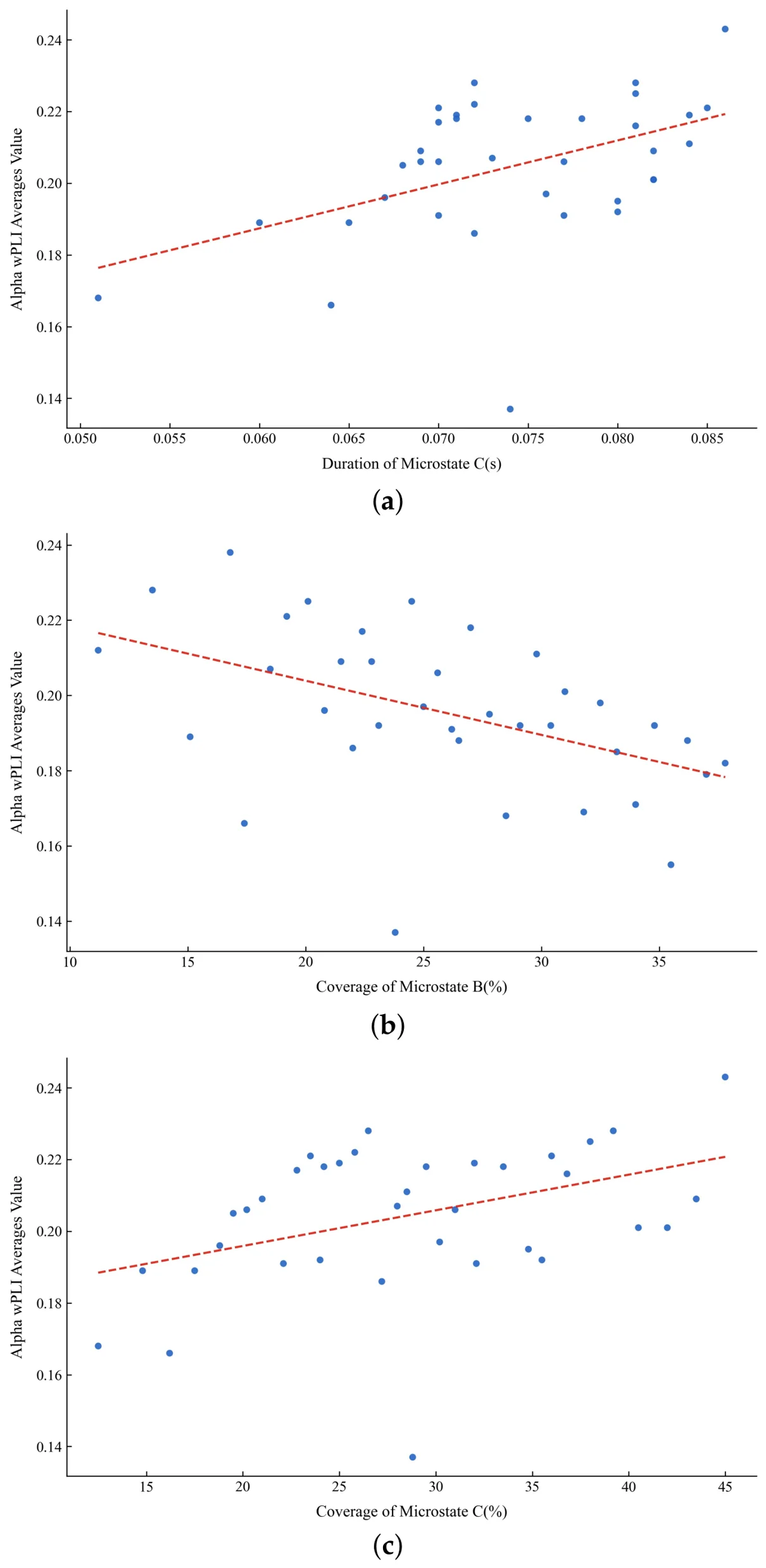
Correlation between microstate parameters and wPLI in the AD group. (**a**) Duration of microstate C was positively correlated with the average alpha wPLI (PCC=0.4664, p=0.0041), (**b**) Coverage of microstate B was negatively correlated with the average alpha wPLI (PCC=−0.4651, p=0.0043), (**c**) Coverage of microstate C was positively correlated with the average alpha wPLI (PCC=0.4137, p=0.0121).

In order to further understand the differences in brain function among different groups, the correlation between the average wPLI value and microstate parameters of each group was analyzed. First, the values of the wPLI matrix and microstate parameters were averaged at the subject level for each group. Pearson correlation analysis was then performed with duration, coverage, and occurrence of the five microstates, with statistical significance evaluated at the standard p<0.05 threshold. Figure 6 demonstrates the correlation between microstate parameters and wPLI in the AD group ($N_{AD}=36$).

Firstly, the duration of microstate C showed a clear positive correlation with the average wPLI values in the alpha band. In both the FTD and HC groups, this correlation was quite similar, with Pearson correlation coefficients (PCC) of 0.6875 and 0.6265, respectively. This suggested that in these two groups, microstate C played a consistent role in brain network connectivity, potentially linked to higher alpha band synchrony. However, in the AD group, this correlation was weaker, with a PCC of 0.4102 (p=0.0129). This indicated that microstate C may have had a diminished role in brain network connectivity or lower synchrony in the alpha band in the AD group, aligning with the cognitive decline observed in AD patients.

Secondly, the coverage of microstate B was negatively correlated with the average wPLI values in the alpha band. The PCCs for the AD, FTD, and HC groups were −0.3482 (p=0.0374), −0.3780, and −0.4990, respectively. This negative correlation indicates that higher coverage of microstate B is statistically associated with lower overall alpha-band wPLI functional connectivity across all groups, with the strongest correlation observed in the HC group (PCC = −0.4990).

Additionally, the coverage of microstate C was positively correlated with the average wPLI values in the alpha band, with the strongest correlation in the FTD group (PCC = 0.7091), followed by the HC group (PCC = 0.5767) and the AD group (PCC = 0.4137, p=0.0121). This indicated that in the FTD group, microstate C played a more crucial role in maintaining brain network synchrony, possibly related to specific neuropathological features of FTD patients.

Notably, negative correlations between the coverage of microstates A, B, and E and the average wPLI values in the alpha band were found only in the FTD and HC groups. In the HC group, these correlations were stronger (APCC = −0.5385, BPCC = −0.5720, EPCC = −0.5027), while they were relatively weaker in the FTD group (APCC = −0.4554, BPCC = −0.4312, EPCC = −0.4149). These negative correlations between alpha-band wPLI and the coverage of microstates A, B, and E were uniquely observed in the FTD and HC groups. Additionally, a positive correlation between microstate D duration and alpha-band wPLI was uniquely observed in the HC group (PCC = 0.4034).

In summary, these findings revealed significant differences in brain network connectivity and microstate dynamics among different groups, particularly between AD, FTD, and healthy individuals. These differences provided new insights and exploratory candidate features for characterizing neurodegenerative conditions.

Furthermore, it was evident in this study that the correlation between the parameters of the microstate and the wPLI matrix was concentrated in the alpha band. This may be related to the fact that this study adopted the paradigm of resting state with eyes closed, while the alpha band (8–12 Hz) is usually associated with people’s relaxed state, eyes closed state, and state of preparation for information processing. This may also be the reason why numerous studies on microstates will filter the signal to 2–20 Hz. Previous studies have suggested that microstate C is associated with large-scale brain networks, whereas alpha-band functional connectivity reflects long-range neural synchronization. Therefore, the observed positive correlation between microstate C duration and alpha-band wPLI may indicate coordinated alterations in temporal brain dynamics and functional connectivity in Alzheimer’s disease. Nevertheless, the underlying neurophysiological mechanism remains to be further investigated.

### 4.5. Analysis of Feature Selection

**Figure 7:**
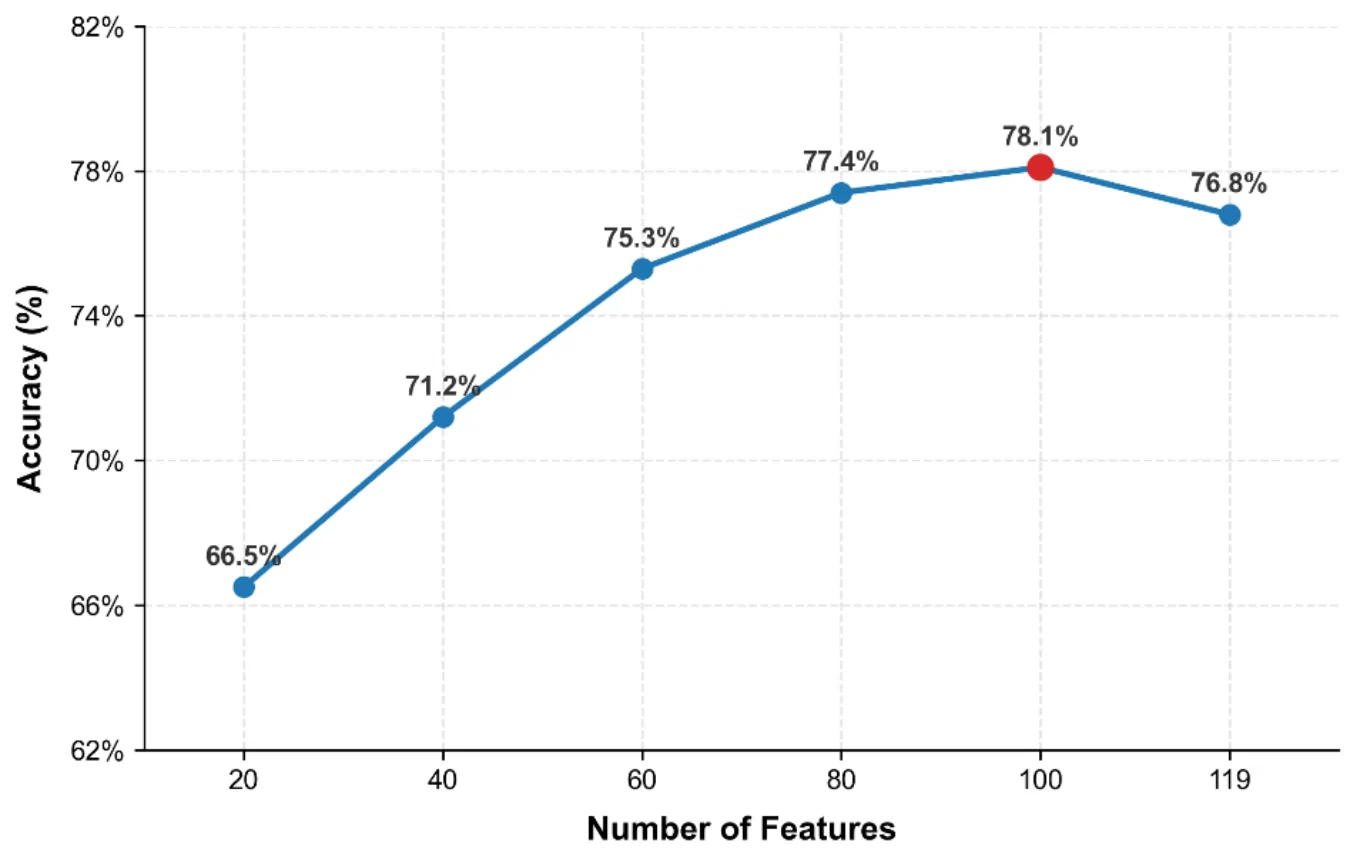
Classification accuracy as a function of the number of selected ReliefF features under subject-independent cross-validation.

It was found that in the classification experiments of AD, FTD and HC, the accuracy reached the highest when the number of features was about 100. Figure 7 shows the classification accuracy of AD, FTD and HC in Dataset I as a function of the number of selected features. Analysis of the 20 features finally selected by the ReliefF algorithm found that these features belonged to the wPLI matrix, and 16 of them were located in the delta band (1–4 Hz), indicating that the connectivity difference across AD, FTD and HC was not too large in this frequency band. In the theta band (4–8 Hz) and beta band (13–30 Hz), there were two features each, and both were the average connectivity features of insula itself and limbic itself. The same two features were also present in the 16 features of the delta band. This indicated that in the three frequency bands, insula and limbic may be less important than other features across the three groups.

### 4.6. The Ability of Combined Features in Predicting MMSE Scores in Patients

To validate the effectiveness of combined features in diagnosing Alzheimer’s disease, predictive models were developed using MMSE scores from both patients and healthy controls. Predictive analyses were conducted separately on individual populations and a mixed population, with a linear regression model serving as the baseline and a KNN regression model used for comparative evaluation.

Mean Squared Error (MSE), Root Mean Squared Error (RMSE), Mean Absolute Error (MAE) and R^2^ were selected as evaluation metrics. The performance of two regression models is presented in Table 7. It can be observed from the table that the KNN model achieves notably better results than the baseline model (linear regression) in every subgroup, indicating its enhanced predictive performance.

Among all subgroups with KNN model, the AD, FTD mixed group achieved the best results (MSE = 1.4775, RMSE = 1.2155, MAE = 0.2169, R^2^ = 0.9245). This may be attributed to the higher homogeneity observed in patients with FTD group compared to those with AD, as FTD group exhibits a more consistent and detectable pathophysiological pattern. In contrast, AD patients may present greater heterogeneity in the underlying pathological mechanisms of neurodegeneration. Furthermore, after incorporating the HC group, the predictive ability of all models declined significantly (MSE = 3.9804, RMSE = 1.9951, MAE = 0.4050, R^2^ = 0.8975). This decline may be due to the distinct biological characteristics between healthy subjects and those in a diseased state, which follow different data distributions. Combining these groups may introduce substantial variability, thereby obscuring disease-specific predictive signals.

## 5. Conclusions

In order to make better use of EEG to diagnose AD, this study proposed a multi-domain feature fusion algorithm that combines the functional connection features of EEG source signals with the microstate features. By extracting the multi-band brain functional connection matrix of EEG source signal and various features of the microstate, the information in spatial, frequency and time domains were well combined and the proposed algorithm achieves excellent classification performance in the OpenNeuro open dataset. In addition, the cross-dataset applicability of the algorithm was preliminarily explored using clinical data from Sir Run Run Shaw Hospital. Moreover, the differences and correlations of each feature in different groups were analyzed in detail so as to understand the pathophysiological mechanism of AD better.

However, the present study still has several limitations. The selected brain region template contained the insula and limbic regions, whereas no corresponding EEG electrodes were available, and alternative brain region templates were not investigated. In addition, although the proposed SMMS framework was evaluated using several conventional machine learning classifiers, its compatibility with neural-network-based classifiers has not yet been systematically explored. Future work will investigate the integration of the proposed SMMS feature representation with representative deep learning classifiers, particularly Multilayer Perceptron (MLP), which is suitable for feature-vector-based classification because it can take the selected SMMS features as input and learn nonlinear decision boundaries through multiple fully connected layers. Moreover, more advanced neural network architectures will be explored when larger datasets become available. Additionally, the identified source connectivity and microstate alterations should be interpreted strictly as exploratory candidate features rather than established biomarkers. Further clinical validation in prospective, independent multi-center cohorts is required to verify their diagnostic utility and generalizability.

## Figures and Tables

**Table 1:** Comparison of representative EEG-based Alzheimer’s disease diagnosis studies.

Method	Task	Accuracy (%)	Spatial Res.	Limitation	Significance
EEG Microstate Analysis	AD vs. HC	85.0	Low	Only temporal dynamics were considered	Demonstrated abnormal microstate dynamics in AD patients
Microstate-Based Brain Network	MCI vs. HC	88.0	Low	No source localization was employed	Integrated microstate information with brain network topology
Frequency-Based FC Network	AD vs. FTD	81.1	Low	Lack of temporal-state information	Revealed discriminative connectivity alterations in dementia
Sleep EEG Connectivity	AD Progression	83.0	Low	Strong dependence on sleep stages	Linked gamma-band connectivity with cognitive decline
SVD Entropy	AD vs. HC	91.0	Low	Limited frequency-domain representation	Validated entropy-based EEG biomarkers for AD diagnosis
CNN + Vision Transformer	AD vs. HC	87.3	Low	Limited interpretability and high data demand	Demonstrated the potential of deep learning for AD diagnosis

**Table 2:** Demographic characteristics of the two datasets (DS II AD indicates the AD groups in Dataset II).

Subjects	Population (Male)	Age	MMSE
AD	36 (13)	66.4±7.9	17.75±4.5
FTD	23 (14)	63.6±8.2	22.17±8.22
HC	29 (11)	67.9±5.4	30
DS II AD	48 (18)	70.4±8.0	18.83±5.2

**Table 3:** Composition of extracted features before and after feature selection.

Feature Category	Description	Number of Features
wPLI features	Upper triangular elements of 6×6 brain-region submatrices across four frequency bands (delta, theta, alpha, beta).	84
Microstate temporal features	Duration, coverage, and occurrence of five microstates.	15
Microstate transition features	Transition probabilities between different microstates.	20
Total extracted features	Combined multi-domain features.	119
Selected features	Features retained by the ReliefF algorithm.	100

**Table 4:** Subject-independent classification performance of SMMS (the row values represent accuracy, precision, recall, and F1-score, respectively).

**Model**	**AD/FTD**	**AD/HC**	**FTD/HC**
KNN	0.842/0.903/0.824/0.862	0.833/0.893/0.806/0.847	0.818/0.881/0.808/0.843
SVM	0.807/0.829/0.853/0.841	0.778/0.762/0.842/0.800	0.826/0.821/0.859/0.840
LDA	0.758/0.821/0.743/0.780	0.730/0.821/0.655/0.729	0.742/0.848/0.663/0.744
DT	0.688/0.746/0.707/0.726	0.717/0.791/0.646/0.711	0.750/0.806/0.707/0.753
Naive Bayes	0.656/0.729/0.671/0.699	0.684/0.763/0.659/0.707	0.743/0.779/0.770/0.775
**Model**	**AD/FTD/HC**	**DS II AD/HC**	
KNN	0.781/0.780/0.788/0.783	0.856/0.779/0.870/0.822	
SVM	0.736/0.737/0.741/0.738	0.832/0.765/0.814/0.789	
LDA	0.684/0.693/0.671/0.681	0.791/0.736/0.747/0.742	
DT	0.652/0.666/0.639/0.651	0.774/0.716/0.726/0.721	
Naive Bayes	0.610/0.642/0.587/0.613	0.736/0.662/0.699/0.680	

**Table 5:** Ablation experiments and comparison under subject-level validation.

Model/Feature Set	AD/FTD	AD/HC	FTD/HC	AD/FTD/HC
Fre-Networks	71.5%	73.2%	72.0%	-
MTRRP	73.0%	75.4%	63.5%	-
SVD	76.5%	75.8%	77.2%	-
ViT-CNN	-	77.5%	74.3%	70.2%
wPLI	68.5%	71.2%	69.4%	60.1%
S-wPLI	76.8%	76.2%	74.5%	70.3%
MS	79.4%	78.5%	76.8%	72.6%
SMMS	84.2%	83.3%	81.8%	78.1%

**Table 6:** Microstate parameters of AD group, FTD group and HC group (Dur indicates duration (ms), Cov indicates coverage (%), Occ indicates occurrence (times/s)) (mean ± standard deviation).

	Ms A	Ms B	Ms C	Ms D	Ms E
Dur	48.7±9.9	54.4±12.9	54.9±16.1	43.8±9.2	51.8±12.6
	44.1±10.6	49.6±9.7	53.2±20.0	41.4±9.4	46.2±11.2
	46.7±8.0	45.4±6.7	60.2±17.9	41.6±7.4	50.4±14.1
Cov	18.2±4.3	24.5±5.9	21.1±8.5	13.5±4.2	22.5±6.8
	18.2±4.9	24.2±6.8	21.2±10.8	14.9±4.7	21.2±6.1
	18.7±5.5	17.5±4.6	27.7±9.2	13.4±3.7	22.5±6.3
Occ	3.8±0.9	4.6±0.9	3.8±1.1	3.1±1.1	4.4±1.1
	4.1±1.1	5.0±1.4	3.9±1.1	3.7±1.3	4.7±1.6
	4.0±1.1	3.9±1.2	4.6±1.0	3.2±0.9	4.5±1.0

**Table 7:** Evaluation metrics for regression models predicting MMSE scores.

	Model	MSE	RMSE	MAE	$R^{2}$
AD	KNN	1.9296	1.3891	0.2537	0.9055
	Linear	14.0372	3.7466	2.9282	0.3127
AD FTD	KNN	1.4775	1.2155	0.2169	0.9245
	Linear	13.0825	3.6170	2.7805	0.3318
AD FTD HC	KNN	3.9804	1.9951	0.4050	0.8975
	Linear	19.3372	4.3974	3.5171	0.5022

## Data Availability

The code used in this study is publicly available at [https://github.com/7788890/SMMS-AD-Code (accessed on 2 April 2026)]. The EEG datasets analyzed in this study are available from OpenNeuro (Dataset I, https://openneuro.org/) and the corresponding author upon reasonable request (Dataset II).
